# Effects of Ginger (*Zingiber officinale,* Roscoe) Essential Oil on Growth and Laying Performances, Serum Metabolites, and Egg Yolk Antioxidant and Cholesterol Status in Laying Japanese Quail 

**DOI:** 10.1155/2019/7857504

**Published:** 2019-03-13

**Authors:** Tchoffo Herve, Kana Jean Raphaël, Ngoula Ferdinand, Ngoumtsop Victor Herman, Ngouozeu Moyo Willy Marvel, Tadondjou Cyril D'Alex, Folack Tiwa Laurine Vitrice

**Affiliations:** ^1^Animal Physiology and Health Research Unit, Faculty of Agronomy and Agricultural Sciences, University of Dschang, P.O. Box 188, Dschang, Cameroon; ^2^Animal Nutrition and Production Research Unit, Faculty of Agronomy and Agricultural Sciences, University of Dschang, P.O. Box 188, Dschang, Cameroon

## Abstract

This study aimed to investigate the effect of ginger (*Zingiber officinale*, Rosc.) essential oil on growth and laying performances, egg yolk antioxidant and cholesterol status, and serum metabolites in Japanese quail. Eighty 3-week-old Japanese quails weighing between 120 and 130 g were equally and randomly assigned to four groups receiving daily and orally, respectively, 100 *µ*l/kg body weight (bw) distilled water and 50, 100, and 150 *µ*l/kg bw of ginger rhizomes essential oil, respectively. The entire feeding trial for all groups lasted for 9 weeks and the* Z. officinale* essential oil effects were studied on growth and laying performances, serum metabolites, and egg yolk antioxidant and cholesterol status. Results revealed that feed intake, live and body weights gain, feed conversion ratio, egg production, and weekly mass of eggs were not significantly (P>0.05) influenced by oral administration of ginger rhizomes essential oil. Unlike the abdominal fat weight which decreased significantly (p<0.05) in all treated quails, the oral administration of ginger rhizomes essential oil had no significant effects (p> 0.05) on liver, intestine, heart, and gizzard relative weights as compared to the control. Egg weight markedly (P<0.05) increased in Japanese quails treated with ginger rhizomes essential oil whatever the dose with reference to the control. The serum content in total cholesterol, LDL-cholesterol, and transaminases (AST and ALT) decreased significantly (P<0.05) with 100 and 150 *µ*l/kg bw of ginger rhizomes essential oil compared to control group. In conclusion, oral administration of 100 to 150 *µ*l/kg bw of ginger rhizomes essential oil to laying Japanese quails positively influences egg weight and decreased serum and egg cholesterols without any adverse effect on feed intake and body weight gain.

## 1. Introduction

Despite scientific progress, the poultry sector still facing managerial and technical problems including poor qualitative and quantitative diet and various diseases that affected growth induced high mortality rates and important economic losses. Among the solutions considered, growth promoters were developed and used to improve feed efficiency and poultry health. Among growth promoters massively used, antibiotics have made a tremendous contribution to the profitability of intensive poultry farming. However, their use as growth promoters has been markedly controversial because of its ability to induce resistance in some pathogenic bacteria strains [1, 2]. This situation has led to the total ban on the use of antibiotics as growth promoters and therapeutic agents in poultry industry. Other food additives such as copper and zinc accumulate in the soil throughout the feces and have a negative impact on the environment because they are not biodegradable [3]. The use of natural compounds that would be available and accessible to all farmers and without prejudice to the environment is topical. As a result, herbal products including essential oils because of their great diversity and diverse biological activities are now obvious [4].

Essential oil is the concentrate and hydrophobic liquid of volatile aromatic compounds [5], such as phenolics and polyphenols, terpenoids, saponins, quinone, esters, flavone, flavonoids, tannins, alkaloids, and nonvolatiles residues. These molecules have many properties including antimicrobial, stimulating animal digestive system, antioxidants, antifungal, antiparasitic, and anti-inflammatory. These properties can reduce loss of energy and improved nutrients absorption for better growth and reproductive performances in animals. Among the aromatic plants containing essential oil is classified the ginger (*Zingiber officinale)*. The ginger, especially the rhizome, contains several biologically active compounds such as gingerol, shogaols, gingerdiol, and gingerdione [6]. These compounds confer to ginger various activities such as antioxidants, antibacterial, anti-inflammatory, antiseptic, antiparasitic, and immunomodulatory properties [7]. Ogbuewu* et al*. [8] reported that ginger rhizomes are good source of micronutrients and pharmacological active compounds that could be useful in animal production to boost growth and reproduction performances. Zhao* et al*. [9] reported laying performances enhancements, serum, and egg antioxidant status in Hy-Line Brown laying hens fed ginger powder during 10 weeks. The same author revealed that ginger powder at the levels of 10 and 15 g/kg of feed increased egg mass and improved serum and yolk antioxidant status. In addition, supplementation of broiler chickens diet with ginger increased total superoxide dismutase and glutathione peroxidase (GSH) activities but reduced malondialdehyde (MDA) and cholesterol concentrations in serum of chickens at 21 and 42 days old [10]. Based on the diversity of active compounds found in ginger essential oil (phenols, terpenes, and alkaloids), we believe that this essential oil could positively influence growth performances and egg yolk status in birds. The objective of this study was to investigate the efficacy of graded levels of ginger rhizome essential oil on growth and laying performances, egg yolk antioxidant and cholesterol status, and serum metabolites in Japanese quail.

## 2. Materials and Methods

### 2.1. Ginger Rhizomes Essential Oil Extraction

Fresh ginger roots were harvested from Santchou (LN 5° 16′ 55^″^, LE 9° 58′ 27^″^) in the Menoua division, West Region of Cameroon. Oil extraction was done by hydrodistillation as described by Wang and Weller [11]. After extraction, the phytochemical screening done according to the methods described by Banso and Ngbede [12] and Ngbede* et al*. [13] showed the results (Table 1).

### 2.2. Animals and Experimental Design

Eighty 3-week-old females Japanese quails (weight: 120-130 g) hatched at the Teaching and Research Farm of the University of Dschang were used for this experiment. Each bird was identified by a ring bearing his number in one of its paws and assigned randomly to 4 dietary treatment groups in a completely randomized design. Each group was divided into 4 replicates of 5 quails. Quails in group 1 (control) received daily by oral way distilled water (100 *µ*l/kg body weight), while birds of the three test groups received, respectively, by the same way 50, 100, and 150 *µ*l/kg bw of ginger roots essential oil. At 12 weeks old, eight female birds per treatment were randomly selected and fasted for 24 hours, weighed, and slaughtered as indicated by Jourdain [14] and blood samples were collected for biochemical analysis. Throughout the experiment,* ad libitum* feed (Table 2) and fresh water were given to all group of birds in adapted equipment. The animal weight was taken at the beginning and weekly to the end of the experiment for the determination of growth performance. This study was carried out at Teaching and Research Farm of the University of Dschang, Cameroon (LN 05°26′, LE 10°3′) and the entire feeding trial for all groups lasted for 9 weeks. The work considered both growing and laying phases. The growing phase lasted 9 weeks while the laying phase was performed at the last 4 consecutive weeks preceding the end of the experiment.

Experimental protocol used in the present study was in accordance with recommendations of institutional guidelines for the care and use of laboratory animals. Quails were humanly handled in respect of the ethical standards laid down in the 1964 Declaration of Helsinki and its later amendments.

### 2.3. Growth Characteristics, Organ Weights, and Laying Performances

The feed intake and the life body weight for individual quail were measured weekly with the scale of 2 kg and 10^−1^ precision; the body weight gain and the feed conversion ratio were determined according to the following formulas:

(1) Body  weight  gain  g=Live  body  weight  of  week  2  g−Live  body  weight  of  week  1  gFeed  conversion  ratio=Weekly  feed  intake  gWeekly  body  weight  gain  g

Organs of sacrificed quails including liver, abdominal fat, intestine, heart, and gizzard were carefully removed and weighed separately with a scale of 160 g and 10^−3^ precision. The relative weight of organs was calculated as follows:

(2) Relative  organ  weight  %=Organ  weight  mgLive  body  weight  g  x100

Egg production and egg weight were recorded daily, during the last 4 consecutive weeks at the end of the experiment.

### 2.4. Blood and Egg Sampling

Blood samples of each slaughtered bird were collected from the jugular vein into tubes free of anticoagulant. After clotting, the serum was separated by centrifugation at 3000 rpm for 15 min and the aliquots were stored at −20°C for biochemical analysis. In addition, the yolk of 12 eggs per treatment was weighted and homogenized with a cold NaCl (0.9%) to obtain a 15% homogenates (yolk weight/NaCl volume). The homogenate was subsequently centrifuged at 3000 rpm for 30 min and the resultant supernatants were kept at −20°C for cholesterol, malondialdehyde (MDA), glutathione (GSH), and catalase (CAT) analysis.

### 2.5. Biochemical Analysis

Serum metabolite (total cholesterol, HDL-cholesterol, triglycerides, transaminases (AST and ALT), and creatinine) contents were measured using methods as described by CHRONOLAB commercial kits. The LDL-cholesterol was calculated as recommended by Friedewald* et al*. [15]. The egg yolk cholesterol level was evaluated using the same method as for serum samples.

### 2.6. Oxidative Stress Characteristics

The yolk content in malondialdehyde was measured by the thiobarbituric acid method [16]. Catalase (CAT) and peroxidase glutathione (GPx) activities were carried out according to the method of Sinha [17] and Ellman* et al*. [18], respectively.

### 2.7. Statistical Analysis

The statistical analysis of the data was performed using the SPSS 20.0 software. The results obtained are expressed as mean ± standard deviation. Differences between groups were assessed using one-way ANOVA followed by Duncan post hoc test. P-value was done using the student t- test. A p value of less than 0.05 was considered as significant. The normality of data was tested by the Shapiro-Wilk Test and the relationships between different parameters highlighted by the correlation coefficient of Bravais Pearson.

## 3. Results

### 3.1. Effects of Ginger Rhizome Essential on Growth Characteristics and Organ Weights in Japanese Quail

As presented in Table 3, the feed intake, the live body weight, the body weight gain, and the feed conversion ratio (FCR) in Japanese quail were not markedly (P>0.05) influenced by the oral administration of ginger rhizomes essential oil, although those growth characteristics tend to decrease in all group of birds treated with ginger rhizomes essential oil compared to the control group.

The oral administration of ginger rhizomes essential oil at all selected doses had no significant effects (p> 0.05) on liver, intestine, heart, and gizzard relative weights as compared to the control. The abdominal fat weight in all treated quails decreased significantly (p<0.05) with reference to that of the control.

### 3.2. Effects of Ginger Rhizome Essential on Serum Biochemical Characteristics and Yolk Oxidative Stress Characteristics in Japanese Quail

The effects of ginger rhizome essential oil on total cholesterol, HDL-cholesterol, LDL-cholesterol, triglycerides, and transaminases are presented in Table 4. As shown in the table, the oral administration of ginger rhizome essential oil at the doses of 100 and 150 *µ*l/kg bw decreased significantly (p<0.05) the serum content in total cholesterol with reference to the control. The serum content in LDL-cholesterol and triglycerides decreased significantly (p<0.05) in all treated birds compared to the control. The serum HDL-cholesterol level increased in all treated groups compared to the control. However, this increase is significant (p<0.05) only at 150 *µ*l/kg bw. The serum content in transaminases (AST and ALT) and creatinine decreased significantly (p<0.05) with ginger rhizome essential oil at all selected doses compared to that of birds in control group. When considering only quails treated with different doses of essential oil, the serum contents in ALT recorded in group of birds exposed at 100 and 150 *µ*l/kg bw were comparable but significantly (p<0.05) decreased with reference to that of birds treated at 50 *µ*l/kg bw. A positive and significant correlation was recorded between the serum content in ALT and the liver relative weight (*ρ* = +0.92; p<0.05) and between the AST level and heart relative weight (*ρ* = +0.95; p<0.05) (Table 6).

The effects of ginger rhizome essential on yolk content in malondialdehyde (MDA) and antioxidant enzymes including peroxidase glutathione (GPx) and catalase (CAT) are shown in Table 4. The yolk MDA concentration decreased significantly (p<0.05) and linearly with the increase of* Z. officinale* essential oil dose with reference to the control. Unlike MDA, the GPx activity increased significantly (p<0.05) and linearly as the essential oil dose increases. Ginger rhizome essential oil at all selected doses increased the CAT activity compared to the control. However, this increase was significantly (p<0.05) only at 100 *µ*l/kg bw.

### 3.3. Effects of Ginger Rhizome Essential on Laying Performances, Yolk Weight, and Yolk Cholesterol in Japanese Quail

As shown in Table 5, ginger rhizomes essential oil at the doses used had no significant (P>0.05) effect on egg production and weekly mass of eggs. The egg weights recorded in all treated groups of quails were increased significantly (p<0.05) compared to that of birds in control group. Yolk weight was not significantly (p>0.05) influenced by the oral administration of ginger rhizomes essential oil. However, it tends to increase whatever the dose of essential oil compared to the control. The yolk weight is positively and significantly correlated to the egg weight (*ρ* = +0.96; P <0.05) (Table 6).

The yolk total cholesterol decreased significantly (p<0.05) with the ginger rhizome essential oil at all selected doses compared to the control. However, between the treated groups, the yolk contents in total cholesterol at 100 and 150 *µ*l/kg bw were comparable and significantly (p<0.05) lower with reference to that of birds treated at 50 *µ*l/kg bw. The total cholesterol per gram of yolk decreased significantly (p<0.05) in groups of Japanese quails exposed at 100 and 150 *µ*l/kg bw of essential oil compared to quails in control group and quails treated with 50 *µ*l/kg bw.

## 4. Discussion

The phytochemical tests carried out in this study reveal that the ginger rhizomes essential oil contains various compounds including phenols, terpenes, and alkaloids. It has been demonstrated that these compounds possess fertilizing properties [19]. In fact, phenolic compounds in general possess various physiological properties such as antioxidant, antiallergic, antiatherogenic, anti-inflammatory, hepatoprotective, antimicrobial, antiviral, antibacterial, anticarcinogenic, antithrombotic, cardioprotective, and vasodilatory [20, 21]. Antioxidant activity prevents the oxidation of low-density lipoproteins (LDL) and limiting their encrustation in the walls of the arteries [20]. In addition, an effective antioxidant system protects embryonic tissue against oxidative responsible for cell death, enhances fertility rate [22, 23] and the survival rate of animals at birth. The alkaloids have the ability to dilate the blood vessels of the sexual organs [24, 25]. Terpenes have antibacterial, antiviral, antifungal and/or anti-inflammatory drugs [26], enabling them to tackle all infections responsible for low fertility in animals.

The oral administration of ginger rhizomes essential oil had no significant effects on feed intake, live body weight, feed efficiency, egg production, and weekly mass of eggs per hen. These results demonstrate that the strongly aromatic odor and pungent taste of ginger oil do not act as a deterrent to feeding. In agreement with the present study, Akbarian* et al*. [27] revealed that feed intake and feed conversion ratio (FCR) were not influenced by the addition of ginger root powder in laying hens feed. Also, Zhang* et al. *[7] reported no significant effects of dietary ginger supplementation (5g/kg) on weight gains of broilers. Contrary, Yahya* et al*. [28] reported that dietary ginger supplementation during the breeding period significantly increased the feed intake and body weight gain in broiler chickens.

The carcass yields recorded in birds treated at 100 and 150 *μ*l/kg bw of essential oil tended to increase compared to that of control birds. These results are close to those of Ngouana* et al*. [29] who found that the use of Oregano essential oil and its combination with thyme oil improved carcass yield compared to the control. Apart from abdominal fat which significantly reduced in treated quails, the relative weights of the liver, the heart, the intestine, and the gizzard were not significantly affected by the essential oil of* Z. officinale* rhizomes. In accordance with this result, El-katcha* et al*. [30] reported that the use of phytobiotics did not significantly influence the relative weight of the different organs. The decrease of abdominal fat recorded in groups of quails exposed to ginger rhizome essential oil would be related to ginger hypolipidemic property. The storage of abdominal fat in animals is controlled by certain enzymes and hormones. Enzymes like lipoprotein lipase hydrolyze VLDL triglycerides and chylomicrons to allow fatty acids to be captured by the adipose cells. On the other hand, monoglyceride lipase promotes the release of fatty acids from storage triglycerides. In addition, some hormones such as insulin promote the fat accumulation. Others like glucagon or adrenaline are lipolytic [31]. The ginger rhizomes essential oil because of these molecules possessing hypolipidaemic properties would increase the activity of lipolytic enzymes or hormones at the expense of enzymes and hormones that favor lipogenesis.

Egg and yolk weights increased in quails exposed to the ginger rhizome essential oil with reference to the control. These results are in agreement to those reported by Arpasova [32] with* Thymus vulgaris *and* Origanum vulgare *essential oil in laying Hy-Line Brown. The increase of egg weight in treated quails is related to the terpenic and phenolic compounds of* Z. officinale* rhizomes. Terpenes due to their antimicrobial property would limit inflammatory reactions costly in energy in favor of egg production. On the other hand, phenolic compounds because of their antioxidant property would limit the alteration of cells or organs involved in the production of eggs and subsequently promote its good formation [33]. A positive and significant correlation was found between the egg and the yolk weights. Based on these results, the egg yolk weight increases as the egg weight increases. The increase in egg component provides sufficient nutrients and centrally placed yolk to support embryonic growth and development [33].

In the present study, the total serum and yolk cholesterol, the LDL-cholesterol, and the triglycerides decreased with oral administration of ginger rhizomes essential oil in Japanese quails. this results are in agreement with the findings of Akbarian* et al*. [27] in laying hens treated with ginger root powder (0.25, 0.5, and 0.75) for 8 weeks and Zeweil* et al*. [34] who noted a significant decrease in LDL-cholesterol, triglycerides, and an increase in blood HDL-cholesterol in Japanese quails treated with ginger rhizomes powder associated with propolis. Ginger rhizomes essential oil exerts it positive effect in animal by interfering with intestinal sterol absorption [35]. Some ginger compounds reduce intestinal reabsorption of biliary cholesterol in laying hens, which modulate whole-body cholesterol in favor of lowering plasma and yolk cholesterol content [27].

Oral administration of ginger rhizomes essential oil in Japanese quails for 12 weeks induced elevation of HDL-cholesterol level. Epidemiological and clinical studies provide evidence that HDL-cholesterol levels are linked to rates of coronary events. This relationship is supported by the potential antiatherogenic properties of HDL, including its mediation of reverse cholesterol transport, in which cholesterol from peripheral tissues is returned to the liver for excretion in the bile [36]. Some studies have suggested that HDL infusions can induce atherosclerosis regression [37]. Protective effect of HDL on atherosclerosis may due to its role in preventing oxidation or other adverse effects of low-density lipoprotein cholesterol (LDL-cholesterol) on endothelial cell. Moreover, HDL can directly stimulate endothelial cell to produce nitric oxide and beneficial anti-inflammatory, antiapoptotic, and antithrombotic agents and promote endothelial repair processes [38, 39].

In the present study, the serum content in AST and ALT was significantly reduced in quail exposed to* Z. officinale* essential oil compared to those of control group. The decrease in AST and ALT levels indicates good liver status. ALT is considered as a useful quantitative marker to describe hepatocellular damage [40]. This enzyme is normally found in the cytosol of hepatocytes and released into blood stream in high level in the case of liver cells membrane impairment. On the other hand, AST is mainly located in the heart and liver cells, and to a lesser extent in the cells of others muscle. Its elevated level in the blood stream is linked to heart or liver cells injury or alteration [40]. The improvement noted here in* Z. officinale* essential oil-treated quails could be explained by the presence in this essential oil and the active biomolecules such as phenols and flavonoids that fight against oxidative stress and subsequently protect the heart and liver cells [41]. Creatinine is a waste produced by protein metabolism in the muscles of animals. It is eliminated by the kidneys and generally used as an indicator of kidney function [41]. Its serum level decreased significantly in treated quails compared to the control group. This could suggest a good function of nephrons. The decrease in plasma creatinine would indicate the increased ability of the kidneys to filter waste from the blood and excrete it in the urine. The phenols and flavonoids of the essential oil due to their structure capable of capturing free radicals would protect the kidneys by avoiding tubular and glomerular cell damage.

The malondialdehyde (MDA) level decreased significantly in Japanese quails treated with ginger rhizomes essential oil. The reverse effect was noted in the antioxidative enzymes (GPx and CAT). The MDA is a major endogenous lipid peroxidation product and, therefore, the extent of lipid peroxidation by reactive oxygen species (ROS) can be monitored by MDA levels [42]. However, animal's diet with antioxidant properties increases the concentration of antioxidants in developing embryonic tissues, reduces their susceptibility to lipid peroxidation [43], and therefore decreases embryonic mortality rate. Akbarian* et al*. [27] mentioned that ginger enhances antioxidant capacity of birds against ROS and reduce plasma MDA level compared to control group as the case in the present study. The ginger rhizomes essential oil riche in phenols with antioxidant activity protects animal cells against detrimental effects of ROS. This reaction would be responsible for MDA low level recorded in this work.

## 5. Conclusion 

The oral administration of ginger rhizomes essential oil in laying Japanese quails for 12 consecutive weeks at doses of 100 and 150 *µ*l/kg bw positively influenced egg weight and yolk antioxidant status. The ginger rhizomes essential oil significantly decreased the serum and the egg yolk contents in cholesterol, with no adverse effect on feed intake and body weight gain in quails. Ginger essential oil may be used in poultry to reduce cholesterol level in bird's products and increase egg weight with a positive effect on his antioxidant status.

## Figures and Tables

**Table 1:** Phytochemical constituents of ginger essential oil.

Constituents	(+) present; (-) absent
Alkaloids	**+**
Triterpenoid	**+**
Steroid	-
Flavonoid	**+**
Phenol	**+**

**Table 2:** Composition and proximate analysis of the experimental diet.

*Constituents*	Amount (kg/100 kg)
Corn	60
Bran wheat	4.5
Soybean meal	22
Fishmeal	4.5
Oeister shell	2
Bone meal	2
Premix 5%*∗*	5
Total	100

*Chemical composition *	

Crude protein (%)	20.15
Metabolizable energy (Kcal/Kg)	2906.80
Calcium (%)	2.03
Phosphorus (%)	1.27
Lysine (%)	0.44
Methionine (%)	0.14
Sodium (%)	0.22

^*∗*^Premix  5%: mixture of vitamins A, B complex, D, K, and E plus iron, Cu, Zn, Se, Mn, methionine, lysine principally and incorporated at 5% in diet.

**Table 3:** Effects of ginger rhizome essential on growth characteristics and organ weightsin Japanese quail.

Parameters	Essential oil doses (*μ*l/kg body weight)				
	Control	50	100	150	P value
*Growth index*	*(n=20)*	*(n=20)*	*(n=20)*	*(n=20)*	
Feed intake (g)	812.50 ± 44.19	805.50 ± 38.17	796.18 ±28.11	788.21 ±40.16	0.66
Live body weight (g)	231.25 ±16.52	226.71 ± 15.11	230.75 ± 36.96	229.65 ± 21.79	0.98
Body weight gain (g)	258.29 ± 131.44	251.86 ± 131.83	250.86 ± 125.68	255.14 ± 122.253	0.99
FCR	3.65 ± 1.18	3.61 ± 1.11	3.62 ± 1.11	3.51 ±1.03	0.99
Slaughter yield (%)	0.85 ± 0.04	0.85 ± 0.05	0.86 ± 0.03	0.86 ±.0.02	0.80
*Organ weights (g/100 g bw) *	*(n=8)*	*(n=8)*	*(n=8)*	*(n=8)*	
liver	1.74 ± 0.65	1.64 ± 0.27	1.57 ± 0.28	1.47 ± 0.11	0.62
Abdominal fat	1.57 ± 0.27^a^	1.31 ± 0.09^b^	1.09 ± 0.09^c^	1.15 ± 0.12^bc^	0.00
Heart	1.22 ± 0.10	1.11 ± 0.07	1.10 ± 0.12	1.13 ± 0.14	0.25
Intestine	2.81 ± 0.35	3.07 ±0.69	3.22 ± 0.61	3.20 ± 0.44	0.54
gizzard	1.82 ± 0.35	1.93 ± 0.22	1.79 ± 0.45	1.84 ± 0.25	0.82

a and b: on the same line, means with the same letter are not significantly different (p> 0.05). n = number of quails.

**Table 4:** Effects of ginger rhizome essential on serum cholesterol, triglycerides, and yolk oxidative stress characteristics in laying Japanese quail.

Parameters	Essential oil doses (*μ*l/kg body weight)				
	Control	50	100	150	P value
*Serum biochemical characteristics *	*(n=8)*	*(n=8)*	*(n=8)*	*(n=8)*	
total cholesterol (mg/dl)	142.51 ± 37.50^a^	132.20 ± 22.21^ab^	108.88 ± 6.60^bc^	100.35 ± 7.00^c^	0.02
HDL cholesterol (mg/dl)	71.67 ± 4.62^b^	83.22 ± 7.89^ab^	88.99 ± 6.76^a^	76.50 ± 12.75^b^	0.02
LDL cholesterol (mg/dl)	70.84 ± 16.61^a^	48.98 ± 12.58^b^	19.88 ± 8.58^c^	23.86 ± 14.77^c^	0.00
Triglycerides (mg/dl)	73.46 ± 10.39^a^	57.18 ± 4.43^b^	56.91 ± 6.49^b^	56.07 ± 6.16^b^	0.00
AST (U/L)	133.72 ± 13.89^a^	101.22 ± 4.29^b^	109.19 ± 6.92^b^	111.78 ± 6.98^b^	0.00
ALT(U/L)	105.25 ± 20.80^a^	98.18 ± 12.71^b^	47.78 ± 14.93^c^	39.55 ± 24.94^c^	0.00
Creatinine (mg/dl)	0.78 ± 0.12^a^	0.55 ± 0.08^b^	0.47 ± 0.04^b^	0.59 ± 0.22^b^	0.00
*Yolk oxidative stress (gram of yolk)*	*(N=12)*	*(N=12)*	*(N=12)*	*(N=12)*	
MDA	0.061 ± 0.01^a^	0.055 ± 0.01^ab^	0.046 ± 0.01^b^	0.044 ± 0,01^b^	0.04
GPx	400.65 ± 56.29^b^	451.06 ± 27.48^ab^	464.54 ± 25.64^a^	476.80 ± 49.01^a^	0.03
CAT	6.31 ± 0.63^b^	6.60 ± 0.82^b^	6.61 ± 0.57^b^	7.56 ± 0.49^a^	0.01

a, b, and c: on the same line, means with the same letter are not significantly different (p> 0.05). n = number of quails; N= number of eggs; p = probability; MDA = malondialdehyde; GPx = peroxidase glutathione; CAT = catalase.

**Table 5:** Effects of ginger rhizome essential on laying performances, yolk weight, and yolk cholesterol.

Parameters	Essential oil doses (*μ*l/kg body weight)				
	Control	50	100	150	P value
*Laying performances*	*(n=20)*	*(n=20)*	*(n=20)*	*(n=20)*	
Egg production (%)	69.04 ± 6.14	66.67 ± 3.89	69.05 ± 8.25	70.24 ± 7.14	0.89
Weekly mass of eggs per hen (g)	70.75 ± 3.68	69.44 ± 2.02	72.69 ± 4.19	69.81 ± 2.48	0.37
Egg weight (g)	12.34 ± 0.15^c^	12.73 ± 0.51^b^	13.31 ± 0.22^a^	12.76 ± 0.21^b^	0.00
*Yolk characteristics*	*(N=12)*	*(N=12)*	*(N=12)*	*(N=12)*	
Yolk weight (g)	3.68 ± 0.33	3.70 ± 0.32	3.84 ± 0.34	3.73 ± 0.36	0.69
Yolk cholesterol (mg/yolk)	155.20 ± 34.07^a^	121.12 ± 15.41^b^	80.35 ± 12.99^c^	90.21 ± 15.38^c^	0.00
Yolk cholesterol (mg/g of yolk)	35.18 ± 3.65^a^	31.58 ± 4.01^a^	23.04 ± 3.31^b^	26.58 ± 3.63^b^	0.00

a, b, and c: on the same line, means with the same letter are not significantly different (p> 0.05). n = number of quails; N= number of eggs.

**Table 6:** Correlations between some organ weights, transaminases, egg, and yolk weights.

*Parameters*	Egg weight	Total cholesterol	Liver weight	Heart weight
Yolk weight	0.97*∗*	-	-	-
Abdominal fat weight	-	0.91*∗*	-	-
AST	-	-	0.57	0.95*∗*
ALT	-	-	0.92*∗*	0.57

*∗* indicates that the correlation is significant at the 0.05.

## Data Availability

The data sets used during the current study are available from the corresponding author on reasonable request.
